# Multifunctional Composite Materials Based on Anion Exchangers Modified with Copper Compounds—A Review of Their Synthesis Methods, Characteristics and Applications

**DOI:** 10.3390/polym15173606

**Published:** 2023-08-30

**Authors:** Elżbieta Kociołek-Balawejder, Ewa Stanisławska, Igor Mucha, Daniel Ociński, Irena Jacukowicz-Sobala

**Affiliations:** 1 Department of Chemical Technology, Wroclaw University of Economics and Business, 53-345 Wrocław, Poland; ewunia17@outlook.com (E.S.); daniel.ocinski@ue.wroc.pl (D.O.); irena.jacukowicz-sobala@ue.wroc.pl (I.J.-S.); 2 Department of Basic Chemical Sciences, Wroclaw Medical University, 50-556 Wrocław, Poland; igor.mucha@umed.wroc.pl

**Keywords:** composite materials, hybrid anion exchanger, copper oxides, metallic copper, antimicrobial agent, As(III) oxidative adsorption, thermal analysis

## Abstract

As copper and its compounds are of fundamental importance for the development of innovative materials, the synthesis of composites intended for water purification was undertaken in which submicron copper containing particles were dispersed within the matrix of a strongly basic anion exchanger, with a macroporous and gel-like structure. Due to their trimethylammonium functional groups, the host materials alone exhibited an affinity to anionic water contaminants and antimicrobial properties. The introduction of such particles as CuO, Cu_2_O, metallic Cu, CuO/FeO(OH), Cu_4_O_3_, Cu(OH)_2_, Cu_4_(OH)_6_SO_4_, Cu_2_(OH)_3_Cl increased these properties and demonstrated new properties. The composites were obtained unconventionally, in ambient conditions, using eco-friendly reagents. Alternative synthesis methods were compared and optimized, as a result of which a new group of hybrid ion exchangers was created (HIXs) containing 3.5–12.5 wt% of Cu. As the arrangement of the inorganic phase in the resin matrix was atypical, i.e., close to the surface of the beads, the obtained HIXs exhibited excellent kinetic properties in the process of oxidation and adsorption of As(III), as well as catalytic properties for the synthesis of triazoles via click reaction, and also antimicrobial properties in relation to Gram-positive *Enterococcus faecalis* and Gram-negative *Pseudomonas aeruginosa* and *Escherichia coli*, preventing biofilm formation. Using thermogravimetry, the effect of the inorganic phase on decomposition of the polymeric phase was evaluated for the first time and comprehensively, confirming the relationship and finding numerous regularities. It was also found that, depending on the oxidation state (CuO, Cu_2_O, Cu), copper-containing particles affected the textural properties of the polymeric phase endowing a tighter structure, limiting the porosity and reducing the affinity for water.

## 1. Introduction

### 1.1. Background

Copper is one of the most valuable and technically useful metals, and has accompanied humans since time immemorial. What is more, the applications of copper are constantly expanding. Copper shows excellent thermal and electrical conductivity, is part of important alloys, and unlike iron does not corrode. It is fundamental for the development of renewable energy systems [1,2]. It can be processed many times without loss of quality and therefore is one of the most important, sustainable and versatile materials. Copper is one of the few metals with antimicrobial properties—bacteria, viruses and fungi inhabiting its surface die. Copper is widely used in agriculture (pesticides), in medicine and pharmacy (anti-inflammatory agents, cancer therapy, wound healing), in separation processes (chiral separation of amino acids) [3,4,5,6].

The development of nanoscience and nanotechnology in recent years has shaped new areas for the use of copper and its compounds in the form of ultrafine particles with specific properties, depending mainly on their size and shape. Due to their extremely small size, high surface energy and large surface area/volume ratio, these particles have different properties than those observed in bulk form. They find various applications in the field of environmental protection as selective adsorbents, visible-light photocatalysts, conventional catalysts and antimicrobials [7,8,9,10,11,12,13,14,15]. With the development of this new area of science, it has become necessary to develop technologies with improved materials, for example to prevent the release of the nanostructure into the treated media (and then into the surroundings). Ion exchangers as supports for the fine particles meet this condition perfectly. At the same time the obtained composites exhibit unique properties that both components do not exhibit separately. The precipitation of Cu-containing particle directly from cupric salt solution is a simple process. However, this reaction conducted within the matrix of polymer—in the solid polymeric phase proceeds differently and strongly depends on the chemical characteristics of the polymer (polymer can be non-polar, or may contain functional reactive groups- cationic, anionic, reductive or oxidative). In the case of environmental applications, such as water purification processes, hydrophilic functional polymers have greater importance since they usually add or enhance the functionality of the obtained composites. For example, ionic functional groups greatly enhance the kinetics of the adsorption process and enable to obtain significantly higher adsorption capacities by preconcentration of the counter ions within the polymeric phase of the composite. That’s why, it is worth making an effort to incorporate copper-containing particles within the polymeric matrix of ion exchangers. Precipitation of such particles into the cation exchanger matrix is relatively a simple process [16,17], whereas into the anion exchanger matrix is challenging, because the positively changed functional groups of anion exchanger show no affinity for cupric cation. In general, hybrid Cu-based heterogeneous nanostructures have novel synergistic properties arising from the integrated interaction between the disparate components, and are the subject of much current interest.

### 1.2. Ion Exchangers vs. Hybrid Ion Exchangers

Synthetic ion exchangers (including cation exchangers and anion exchangers) are polymers in the form of spherical beads with a cross-linked porous skeleton and ionogenic functional groups capable of an ion exchange reaction. Ion exchangers have been produced for a long time on a mass scale and are used in universal industrial water treatment processes such as demineralization, deionization, softening, decarbonization and dealkalization, conducted in conventional and nuclear power plants and production processes using water as a reagent or reaction medium. In different industrial processes, they are used to improve the quality of the liquid phase by removing undesirable impurities (purification) and to separate valuable substances from solutions (recovery). They are helpful in the preparation of high-quality water (e.g., ultrapure water for electronics) and for the purification of wastewater [18,19,20].

Ion exchangers are not always able to remove certain ions from water, especially if they are present in a low concentration. This applies to various types of micropollutants, which are technologically undesirable/burdensome and can also pose a threat to human health. Why is the traditional ion exchange process ineffective for the removal of various ionic micropollutants from water? A feature of ion exchangers is limited selectivity, which means that it is difficult to isolate ions occurring in low concentrations from the aqueous phase if other ions in higher concentrations are also present, especially ions with higher valence. This can be illustrated by fluoride ions, which are present in water in low concentrations and are harmful to humans [21]. Anion exchangers bind fluoride ions with difficulty for several reasons. The affinity of a given ion to anion exchanger is influenced by a few factors, such as ion valence (affinity increases with the charge of the ion), ion diameter and the strength of the acid from which this ion comes. All these factors are unfavorable for fluoride because it is monovalent and highly hydrated, and hydrofluoric acid is a weak acid (the weakest in the series of HF < HCl < HBr < HI acids), hardly donating protons and therefore weakly dissociated. As a result, $F^{-}$ has the lowest affinity for anion exchangers of all anions, including common monovalent ones occurring in natural waters. Ion exchange using the traditional approach, such as the removal of anions with commercially available ion exchangers, is irrelevant in the case of fluorides.

Extensive use of ion exchangers to remove micropollutants from water, but in a new role, became possible thanks to the development of hybrid ion exchangers (HIXs), which show higher selectivity and higher affinity towards the constituents of water than native ion exchangers. In this role, ion exchangers function as polymeric support for nanoparticles, which show different useful properties as sorption, redox, magnetic, catalytic and biocidal [22,23,24,25]. Although as reactants NPs alone have many advantages, in water they aggregate (which reduces the reaction surface), can only be used in batch systems, and it is difficult to isolate them from the aqueous phase. Separation methods such as magnetic separation, crossflow membrane filtration and centrifugation prolong the purification process, require special equipment and generate costs [26,27,28]. Due to the extremely small dimensions of NPs, at various stages of use they can penetrate in an uncontrolled way into all the spheres of the environment, as well as into the human body. Their application and disposal can have hard-to-predict ecological consequences [29,30].

HIXs are polymeric/inorganic composites in which the inorganic component is metal, metal oxide or metal hydr(oxide) nanoparticles (NPs) dispersed in the matrix of the ion exchanger. Commercially available ion exchangers in the form of water-swelling spherical beads provide easily accessible support for immobilizing NPs within the polymer phase. Due to the mechanical strength, porosity, permeability and the excellent hydraulic characteristics of ion exchangers, it is possible to conduct technological processes using HIXs in a column, which results in numerous technical and environmental benefits. The essence of the synthesis of HIXs is the binding of the precursor, proper cation or anion by ionogenic functional groups in an ion exchange reaction, followed by inorganic deposit precipitation in the skeleton of the resin beads using a solution of suitable precipitating agents (by alkalization or oxidation/reduction reaction). NPs dispersed in the polymeric phase should have appropriate and beneficial physical and chemical properties, that is they must be practically insoluble in water within the required pH range, and must also show adequate reactivity. Several nanostructures containing polyvalent metals such as Fe, Mn, Zr are the most common adsorbents under study [31,32,33,34,35,36,37,38]. There are also HIXs containing zero valent (metallic) deposits such as Fe, Pd, Ag with reducing and catalytic properties [39]. Once immobilized in the resin matrix, NPs do not significantly aggregate or coalesce, that is the surface area available for sorption/reaction does not diminish with prolonged use. HIXs exhibit extended functionality due to the presence of both functional groups, which undergo coulombic interactions and inorganic deposit, allowing a large area of contact between the reagents and offering new separation opportunities involving Lewis’s acid-base interaction. The presence of NPs does not interfere with the functional groups of the ion exchanger, thus providing an opportunity for a synergy not available otherwise [19].

HIXs exhibit a characteristic property called the Donnan membrane effect, in that the ion exchanger functional groups can facilitate (or hinder) the sorption of a particular water constituent [40]. For example, the presence of electropositive functional groups in the anion exchanger favours the migration of the anions present in water deep into the polymeric phase, enabling their sorption into NPs. So HIXs with basic functional groups are useful for the removing the anionic impurities from water. The most important direction of the practical use of HIXs so far is related to this issue. HIXs containing submicron or nanoscale hydrated iron(III) oxide particles in the anion exchanger matrix have proved to be an exceptionally useful sorbent for removing arsenic(V) ions from water [41]. The pollution of natural waters with arsenic is a problem in many regions of the world, threatening millions of people, as inorganic arsenic compounds are highly toxic. One of the major global environmental challenges is to provide potable water with an arsenic concentration below 10 µg/dm^3^. Since arsenic in natural waters occurs in a low concentration compared to common anions, sorption methods are of predominant importance in its removal, and HIXs are ideal for this [42]. An example of commercial success are FerrIX A33E (Purolite, PA, USA) and Seplite LAR714 (Sunresin, Shaanxi, China), which are highly porous hybrid ion (anion) exchange resins infused with iron oxide for efficient, selective removal of arsenic. Over the years, different composite materials obtained using ion exchangers have been described, with the largest share of sorbents obtained with the use of anion exchangers containing FeO(OH), Fe_3_O_4,_ MnO_2_, ZrO_2_, showing sorption affinities toward environmentally significant target ligands and organic contaminants.

### 1.3. Hybrid Ion Exchanger Development Opportunities

Nowadays, for environmental and technological reasons, simple, effective, green (organic solvent free) methods for separation and recovery different ions/compounds from aqueous solutions are needed [43,44,45,46]. Composite materials as sorbents of target pollutants are one of the most popular areas of modern research related to water purification. The literature describes various biochar-based and carbon sorbents from spent materials, which are effective, easily available and cheap reagents. They are characterized by a specific chemical structure/composition and specific properties, which result from their chemical structure, origin and transformations [47,48,49]. HIXs obtained based on commercial ion exchangers are universal materials with a repeatable and reproducible chemical structure/composition, and can act as chemical reagents. In terms of the type of ion exchanger (cation exchanger or anion exchanger), the type of functional groups (strongly acidic, strongly basic, weakly acidic, weakly basic), the different structure of the resin skeleton (macroreticular or gel-like), as well as the type of NPs, there is no doubt that these materials can be numerous and can exhibit different properties. There is still a great deal of potential for the use of HIXs for removing heavy metals from water. Metals such as Hg, Pb and Cd are extremely poisonous for living organisms, and it is important to remove them from water and achieve their ultralow concentrations [50,51,52,53]. According to the standards in EU countries, the maximum permissible concentration of Hg(II), Pb(II) and Cd(II) in water intended for human consumption is as low as 1.0, 5.0 and 10.0 μg/L [54]. To achieve such purification efficiency, HIXs showing above-average affinity to these metals are needed. Many examples can be given of NPs that come under consideration and which may be embedded in appropriate anionic or cationic support. The choice of the functional groups of the polymeric host materials can be harnessed to alter (enhance or diminish) the intrinsic properties of the nanomaterials. By aptly choosing the type of functional groups, the sorption and separation processes can be effectively controlled. The functional groups may also have specific catalytic, stabilizing or biocidal properties (in addition to NPs activity).

## 2. Anion Exchangers as Support for Cu-Based Particles

Due to the environmental potential of metallic copper and copper compounds, HIXs have been developed in which a dispersed phase of copper-containing NPs is embedded in the anion exchanger phase. As polymeric support, two strongly basic anion exchangers were used as they are commercially available and universal in terms of application (Amberlite IRA900 and Amberlite IRA402, produced by The Dow Chemical Company, Midland, MI, USA. There are two types of strongly basic anion exchangers that differ in the structure of the polymeric skeleton, which is a copolymer of styrene and divinylbenzene. In a macroreticular anion exchanger (M, Amberlite IRA900), the skeleton contains macropores and exhibits a large inner surface, so large molecules can move freely in the resin into the centre of a bead. In contrast to macroreticular anion exchanger, in a more compact gel-like structure anion exchanger (G, AmberliteIRA402) the skeleton is devoid of real pores, as a result of which functional groups in the interior of the bead may be hard to reach for large molecules. Both types of anion exchangers contain trimethyl ammonium functional groups ($-N{\left({\mathrm{CH}}_{3}\right)}_{3}^{+}$) with a similar ion exchange capacity, in the order of 3.2 meq/g. They also have the limited thermal resistance, with a maximum operating temperature of 60 °C in the ${\mathrm{OH}}^{-}$ form and 75 °C in the ${\mathrm{Cl}}^{-}$ form.

Considering the multifunctionality of hybrid ion exchangers, the choice of anion exchangers as a supporting material was a very promising challenge. This choice was particularly interesting due to the presence of quaternary ammonium groups, which may not only be involved in the preconcentration of negatively charged reagents in the resin phase, but also show antimicrobial activity (the sulfonic and carboxylic functional groups of cation exchangers do not exhibit such a property), and can enhance the biological efficiency of the obtained composites [55,56,57,58].

It is worth mentioning that a study published in 1949 by F.G. Mills and N.B. Dickinson [59] is considered to be the first study on hybrid ion exchangers, and interestingly these were copper containing HIXs. More than 70 years ago, it was shown that an anion exchanger containing dispersed “colloidal copper” was capable of removing dissolved oxygen from water either in a columnar operation or in a batch process:

(1) $$2{\mathrm{Cu}}^{0}+O_{2}+4H^{+}\to 2{\mathrm{Cu}}^{2+}+2H_{2}O$$

In a two-step synthesis procedure, a weakly basic anion exchanger containing amine groups formed very poorly dissociated complexes with copper salts:

(2) $$4{\mathrm{RNH}}_{2}+{\mathrm{CuSO}}_{4}\to [\mathrm{Cu}\left({\mathrm{RNH}}_{2}{)}_{4}\right]{\mathrm{SO}}_{4}$$

Then, the blue green in the colour complex was reduced with an alkaline solution of sodium hydrosulfite, yielding a deep purple precipitate containing metallic copper (extremely susceptible to oxidation):

(3) $${\mathrm{CuSO}}_{4}+{\mathrm{Na}}_{2}S_{2}O_{4}+2H_{2}O\to {\mathrm{Cu}}^{0}+{\mathrm{Na}}_{2}{\mathrm{SO}}_{4}+2H_{2}{\mathrm{SO}}_{3}$$

In the process taking place in the column, some colloidal copper and/or cuprous oxide appeared in the effluent. In addition, small amounts of metal were leached from the resin during regeneration. This meant that the precipitate passed to some extent from the resin phase to the aqueous phase. Prevention of such phenomena is one of the most important problems to be surmounted in the synthesis of modern HIXs.

**Figure 1:**
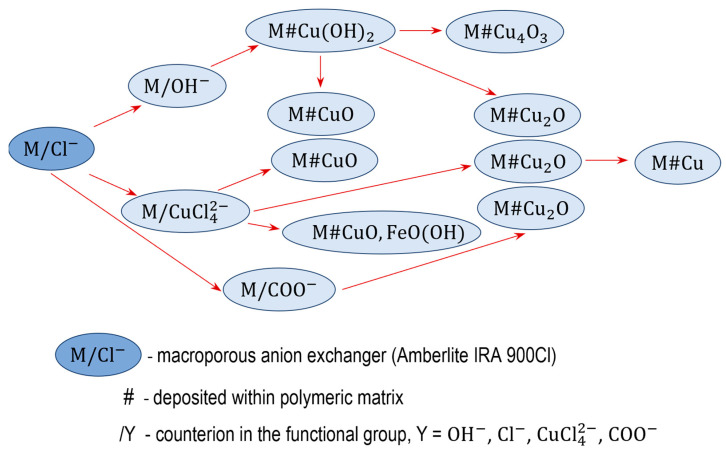
Transformations of macroporous anion exchanger.

**Figure 2:**
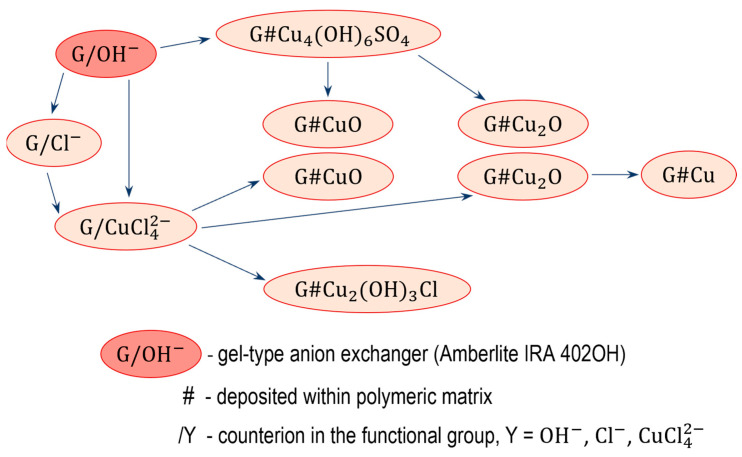
Transformation of gel-type anion exchanger.

The electropositive functional groups of the anion exchangers are not able to bind copper cations from copper salt solutions, they repel ${\mathrm{Cu}}^{2+}$ ions. Therefore, using the anion exchangers as a supporting material is challenging and requires an unconventional approach (Figure 1 and Figure 2). Two routes lead to the preparation of anion exchanger/copper containing NP composites: indirect, in which the anion exchange functional groups do not attach ions containing a copper atom at any stage, and direct, in which the anion exchange functional groups attach ions containing a copper atom.

## 3. Methods of Introduction of Cu-Based Particles into Anion Exchangers

### 3.1. Indirect Method of Synthesis of a Macroporous Anion Exchanger as Support for Cu(OH)_2_, CuO, Cu_2_O and Cu_4_O_3_

The transformations (4) were performed using a macroporous anion exchanger (M) as polymeric support [60]. The introduction of copper-containing particles such as Cu(OH)_2_, CuO, Cu_2_O and Cu_3_O_4_ into the resin phase occurred with the use of ${\mathrm{Cu}}^{2+}$ ions contained in the aqueous phase.



(4)

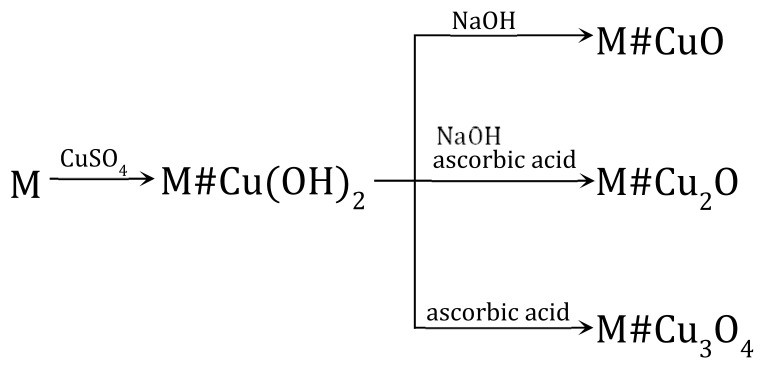




An anion exchanger whose functional groups were conducted from the ${\mathrm{Cl}}^{-}\;$ in the ${\mathrm{OH}}^{-}$ form, was reacted with 0.1 M CuSO_4_. Crucial for the efficient deposition of cupric compounds within the polymeric matrix was an optimal dose of CuSO_4_ used as the reagent—slightly exceeding (no more than 15–20%) the stoichiometric dose resulting from the reaction Equation (5). The source of ${\mathrm{OH}}^{-}$ ions needed to produce Cu(OH)_2_ were the functional groups of the anion exchanger:

(5) $$2\left[P\right]-N^{+}{\left({\mathrm{CH}}_{3}\right)}_{3}{\mathrm{OH}}^{-}+{\;\mathrm{CuSO}}_{4}\to {\left(\left[P\right]-N^{+}{\left({\mathrm{CH}}_{3}\right)}_{3}\right)}_{2}{\mathrm{SO}}_{4}^{2-}\#\mathrm{Cu}{\left(\mathrm{OH}\right)}_{2}$$

[P] is the polymer matrix (styrene-divinylbenzene copolymer).

Cupric hydroxide, a blue water-insoluble sediment, was precipitated inside the beads as a result of the ion exchange reaction. The reaction proceeded smoothly at an ambient temperature because the functional groups of the anion exchanger show a high affinity for ${\mathrm{SO}}_{4}^{2-}$ ions, and since the ${\mathrm{OH}}^{-}$ ions released from functional groups alkalized the reaction medium (pH about 5.5), Cu(OH)_2_ could precipitate (6):

(6) $${\mathrm{Cu}}^{2+}+2{\mathrm{OH}}^{-}\to \mathrm{Cu}{\left(\mathrm{OH}\right)}_{2}$$

Most of the precipitate (about 70%) remained in the anion exchange skeleton, while the rest passed into the aqueous phase. M#Cu(OH)_2_ contained nearly 7.2 wt% of Cu. Cu(OH)_2_ was deposited mainly in the outer parts of the beads, while the inner parts were covered to a much lesser degree. The uneven deposition of cupric hydroxide within the interior of the beads was the result of the presence of functional groups which, due to the co-ion exclusion effect, constrict the penetration of ${\mathrm{Cu}}^{2+}$ ions within the polymeric matrix.

M#Cu(OH)_2_ served as an intermediate product to introduce CuO into the matrix of the anion exchanger [60] as a result of the following transformation (7):

(7) $$M\#\mathrm{Cu}{\left(\mathrm{OH}\right)}_{2}\overset{\mathrm{NaOH}}{\to }M\#\mathrm{CuO}+H_{2}O$$

For the course of the reaction (7) it was important that from Cu(OH)_2_ one can obtain CuO in relatively mild conditions because anion exchangers have low thermal resistance. To carry out this transformation effectively, it was necessary to introduce the semi-finished product into a NaOH solution at 50 °C and keep it at this temperature briefly (for nearly 5 min). The M#CuO contained 5.4 wt% of Cu. The CuO particles were accumulated close to the surface of the beads. The following transformation: M→$M\#\mathrm{Cu}{\left(\mathrm{OH}\right)}_{2}$→M#CuO was repeated on the anion exchanger three times, whereby it was possible to introduce additional portions of CuO into the polymeric matrix—8.5 wt% of Cu after the second cycle and 12.2 wt% of Cu after the third cycle. However, after the third cycle, the beads were partially cracked due to the increasing and unevenly deposited CuO load.

M#Cu(OH)_2_ also served as a semi-finished product to introduce Cu_2_O into the matrix of the anion exchanger [61] to obtain M#Cu_2_O (8):

(8) $$M\#\mathrm{Cu}{\left(\mathrm{OH}\right)}_{2}\overset{\begin{array}{c} \mathrm{ascorbic}\;\mathrm{acid}\\ \mathrm{NaOH} \end{array}}{\to }M\#{\mathrm{Cu}}_{2}O$$

The Cu(OH)_2_ deposit was converted into Cu_2_O through a reduction reaction through contact with a solution of ascorbic acid (AA) or glucose (GL). Both reducers made it possible to obtain HIXs with a high Cu_2_O content in mild conditions, but the HIX with the highest Cu_2_O content (up to 7.0 wt% of Cu) was obtained by reduction in an AA/NaOH medium at an ambient temperature for 24 h. Even though it was apparent that the reduction on the surface of the beads proceeded rapidly (faster with ascorbic acid than with glucose), it was ascertained that it was necessary to conduct the reaction for many hours in order for the Cu(OH)_2_ contained deep in the beads to be transformed into Cu_2_O:



(9)

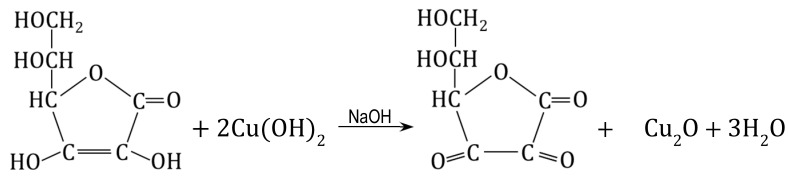




In the reaction of M#Cu(OH)_2_ with the solution of ascorbic acid alone, a mixed deposit containing slight Cu(0), Cu(I) and Cu(II) was precipitated in the anion exchanger matrix, in which paramelaconite was unexpectedly identified. By chance, the selected reaction parameters created conditions favorable to the formation of such a unique copper oxide, as a metastable, mixed valence oxide in which the copper is present in both oxidation states: Cu(I) and Cu(II) (Cu_4_O_3_) (10):

(10) $$2\mathrm{Cu}{\left(\mathrm{OH}\right)}_{2}+{\mathrm{Cu}}_{2}O\to {\mathrm{Cu}}_{4}O_{3}+2H_{2}O$$

It is worth emphasizing that in the case of identifying a multi-component deposit, its presence in the resin matrix was an advantage and facilitated the analysis process. HIX offers the possibility of investigating the deposit composition using leaching procedures with different eluents (different acids of different concentrations, concentrated aqueous ammonia).

A study on another group described a HIX containing dispersed Cu_2_O particles stabilized onto the anionic resin surface [62]. An anion exchanger in the formate form was reacted with cupric acetate in DMF at 120 °C. The counter formate anions (${\mathrm{HCOO}}^{-}$) reduced precursor Cu(II) to Cu(I). The resulting M#Cu_2_O contained 1.1 wt% of Cu. The authors did not comment on the effect of such a high reaction temperature on the stability of the polymeric support.

### 3.2. Indirect Method of Synthesis, Gel-like Anion Exchanger as Support for Cu_4_(OH)_6_SO_4_, CuO and Cu_2_O

The transformations (11) were performed using as the polymeric support a gel-like anion exchanger (G). The introduction of copper-containing particles such Cu_4_(OH)_6_SO_4_, CuO and Cu_2_O into the resin phase produced Cu^2+^ ions contained in the aqueous phase.



(11)

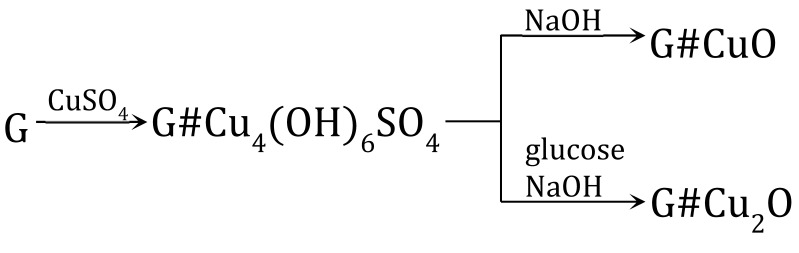




An anion exchanger in the ${\mathrm{OH}}^{-}$ form was reacted with 0.1 M CuSO_4_ analogous to reaction (5), but using G as polymeric support [63]. Although the procedure was the same as before, in the case of G it was not cupric hydroxide, but the rare and peculiar deposit cupric hydroxysulfate (brochantite) formed in the resin phase and coloured the beads in celadon. It turned out that the gel-type matrix of the resin was crucial to the pathway of cupric compound deposition. The same procedure of Cu(II) compound deposition within the gel-type polymeric matrix of the anion exchanger led to other products due to the different course of reaction (5) resulting from the hindering effect of the polymeric structure. Such a deposit as brochantite precipitated into the matrix of the anion exchanger because the ion exchange reaction was slow due to the impeded access of ${\mathrm{SO}}_{4}^{2-}$ ions (because of the gel matrix) to the active site of the anion exchanger. As G reacted with the CuSO_4_ solution slowly, the ${\mathrm{OH}}^{-}$ ions entered the reaction gradually (this slowed down the increase in pH of the reaction medium, which did not exceed 4.8), and was advantageous for the formation of brochantite (5), (12), (13).

(12) $${\mathrm{CuSO}}_{4}+3\mathrm{Cu}{\left(\mathrm{OH}\right)}_{2}\to {\;\mathrm{Cu}}_{4}{\left(\mathrm{OH}\right)}_{6}{\mathrm{SO}}_{4}$$

(13) $$3{\left(\left[P\right]-N^{+}{\left({\mathrm{CH}}_{3}\right)}_{3}\right)}_{2}{\mathrm{SO}}_{4}^{2-}\#\mathrm{Cu}{\left(\mathrm{OH}\right)}_{2}+{\mathrm{CuSO}}_{4}\to {\left({\left(\left[P\right]-N^{+}{\left({\mathrm{CH}}_{3}\right)}_{3}\right)}_{2}{\mathrm{SO}}_{4}^{2-}\right)}_{3}\#{\mathrm{Cu}}_{4}{\left(\mathrm{OH}\right)}_{6}{\mathrm{SO}}_{4}$$

Since the formation of Cu_4_(OH)_6_SO_4_ involved part of ${\mathrm{SO}}_{4}^{2-}$ ions introduced into the reaction medium, transformation of the functional groups from the hydroxyl to sulphate form was not completed. In consequence, the theoretical Cu load was less than the anion exchange capacity of the G. The G#Cu_4_(OH)_6_SO_4_ contained 6.0 wt% of Cu, constituting approximately 82% of the theoretically introducible amount.

The transformation of Cu_4_(OH)_6_SO_4_ into CuO within the gel-type matrix was a troublesome and inefficient transformation because the deposit left the resin phase (passed into the aqueous phase) [63]. The gel-type anion exchanger contained the highest CuO load (2.9 wt% of Cu) when the G#Cu_4_(OH)_6_SO_4_ was alkalized at an ambient temperature in a wet state (these conditions limited swelling and deformation of the polymeric skeleton, and crumbling of the inorganic deposit). Nevertheless, conversion of Cu_4_(OH)_6_SO_4_ into CuO proceeded with the highest yield of 45%.

The Cu_4_(OH)_6_SO_4_ deposit in a gel-type anion exchanger can be transformed into Cu_2_O through a reduction reaction [64]. Ascorbic acid and glucose in an NaOH solution were used as reducers. Both reactants reduced the brochantite, but in contrast to reaction (9), ascorbic acid yielded abundant Cu_2_O outside the resin phase. G#Cu_2_O with the highest Cu_2_O content (3.0 wt% of Cu) was obtained in the reaction involving glucose, conducted for 3–4 h at an ambient temperature (14):

(14) $$2{\mathrm{Cu}}_{4}{\left(\mathrm{OH}\right)}_{6}{\mathrm{SO}}_{4}+3{\mathrm{CH}}_{2}\mathrm{OH}{\left(\mathrm{CHOH}\right)}_{4}\mathrm{CHO}\overset{\mathrm{NaOH}}{\to }\;\to 3{\mathrm{Cu}}_{2}O+3{\mathrm{CH}}_{2}\mathrm{OH}{\left(\mathrm{CHOH}\right)}_{4}\mathrm{COOH}+2{\mathrm{CuSO}}_{4}+6H_{2}O$$

The HIX obtained in the same conditions but involving ascorbic acid contained half as much copper. It turned out that the type of reducer had the greatest effect on the forming Cu_2_O remaining in the anion exchange phase.

### 3.3. Comments on Indirect Synthesis

Summing up this part of the synthesis, it can be stated that using both types of anion exchangers (M and G) and a CuSO_4_ solution, different copper-containing HIXs can be obtained. Using an unconventional method of synthesis, common reactants, and mild reaction conditions, HIXs can be obtained containing quite a large amount of copper deposit, constituting about 50–70% of the theoretically introducible amount. Of interest were the results of two syntheses, which showed a significant influence of the resin skeleton structure on the course of the reaction (5), when different HIXs (M#Cu(OH)_2_ vs. G#Cu_4_(OH)_6_SO_4_) are formed in the same reaction conditions [60,63]. The choice of reagents (ascorbic acid/M#Cu(OH)_2_ vs. glucose/G#Cu_4_(OH)_6_SO_4_) in the synthesis of Cu_2_O was also interesting, as it transpired that the type of reductant (salt vs. non-ionic compound) significantly influenced the reaction course (Cu_2_O inside the resin phase vs. Cu_2_O outside the resin phase) [61,64]. It also turned out that parameters usually beneficial for the course of the reaction, such as an excess of reactant, longer reaction time and increased temperature, may adversely affect the content of the deposit in HIXs. It was also interesting that some subtle actions such as drying the resin sample before the reaction (this concerned reaction (5)), or conversely, the use of the resin sample in a wet state (this concerned the transformation of G#Cu_4_(OH)_6_SO_4_ to G#CuO) may be crucial for beneficial conversion. These details allow it to be concluded that it is more difficult to obtain HIXs than NPs alone (without a polymeric carrier) because the key problem is to select such reactants and reaction parameter values which would ensure that the forming particles remained in the resin phase. It is worth noting that the atypical deposition of cupric compounds (Cu(OH)_2_, CuO, Cu_4_(OH)_6_SO_4_) in the outer parts of the beads can be advantageous considering the kinetics (limiting the influence of diffusion) of reactions that proceed with their use (adsorption, photocatalytic reactions, transformations of cupric deposit, antimicrobial action).

### 3.4. Direct Method of Synthesis, Transformation of Anion Exchangers in the $CuCl_{4}^{2-}$ Form

Anion exchanger/copper composites containing NPs can also be obtained by the direct method, the basis of which is the attachment of an anion containing copper to the anion exchanger functional groups. The unique and only possible anion is ${\mathrm{CuCl}}_{4}^{2-}.$ The tetrachlorocuprate(II) ion exists only in the aquatic media under strictly defined conditions. It is formed in a cupric chloride solution in the presence of an excess amount of hydrochloric acid or chloride salt (15):

(15) $${\mathrm{CuCl}}_{2}+2{\;\mathrm{Cl}}^{-}\;\to {\mathrm{CuCl}}_{4}^{2-}$$

The basis of the proposed method was to determine the composition of the solution allowing for the quantitative transformation of the anion exchanger functional groups in the ${\mathrm{CuCl}}_{4}^{2-}$ form. Concentrations of both reagents CuCl_2_/NaCl and CuCl_2_/HCl for transforming the functional group from the ${\mathrm{Cl}}^{-}$ into ${\mathrm{CuCl}}_{4}^{2-}$ form were sought (16):

(16) $$2\left[P\right]-N^{+}{\left({\mathrm{CH}}_{3}\right)}_{3}{\mathrm{Cl}}^{-}+{\;\mathrm{CuCl}}_{4}^{2-}\to {\left(\left[P\right]-N^{+}{\left({\mathrm{CH}}_{3}\right)}_{3}\right)}_{2}{\mathrm{CuCl}}_{4}^{2-}+2{\mathrm{Cl}}^{-}$$

It was found that reaction (16) proceeded quantitatively and quickly (in few minutes) in batch conditions when the reaction medium was 0.5 M CuCl_2_ in 5 M NaCl or 0.5 M CuCl_2_ in 5 M HCl (the solution turns green when the ${\mathrm{CuCl}}_{4}^{2-}$ ion is formed) [65]. The reaction medium, a concentrated NaCl solution or concentrated HCl solution, had no significant effect on the course of the reaction, as both reaction media were highly acidic (the pH of 0.5 M CuCl_2_ in 5 M NaCl was 2.5). When the reaction medium was a solution with a lower chloride concentration, for example 0.5 M CuCl_2_ in 2 M NaCl, then only about 30% of the functional groups passed into the ${\mathrm{CuCl}}_{4}^{2-}$ form. Importantly, as the ${\mathrm{Cl}}^{-}$ ion concentration in the solution is decreased or the pH is increased, the ${\mathrm{CuCl}}_{4}^{2-}$ ion desorbs from the resin and decomposes to positively charged Cu(II) species. Given the limited stability of the ${\mathrm{CuCl}}_{4}^{2-}$ ion, the sample of resin after the reaction could not be washed with deionized water since this would immediately result in the release of copper from the functional groups to the water phase. After the reaction the samples were vacuum filtered (using a Büchner funnel) to precisely separate (as far as possible) the post-reaction medium from them. Owing to the vacuum filtration (unlike gravitational filtration), the intermediate products had a low moisture content (about 50%), thanks to which the next reagents could penetrate faster into the anion exchanger beads.

Working with the functional groups of the gel-like anion exchanger in the ${\mathrm{CuCl}}_{4}^{2-}$ form was more difficult [66]. In a gel resin (without artificial open pores), the functional groups in the interior of the bead are harder to reach for a large molecule such as the ${\mathrm{CuCl}}_{4}^{2-}$ ion. When 0.5 M CuCl_2_ in 5 M HCl was used as a reaction medium (because the commercial resin was in the ${\mathrm{OH}}^{-}$ form), the ion exchange reaction did not proceed very fast nor in significant quantities, as after 2 h of shaking only 75% of the functional groups were transformed into the ${\mathrm{CuCl}}_{4}^{2-}$ form. The cause of incomplete conversion could be the shrinkage of the resin (by about 30%) during the transformation of the ionic form of the functional groups from ${\mathrm{OH}}^{-}$ to ${\mathrm{Cl}}^{-}.$ When the reactants were changed: using the anion exchanger in the ${\mathrm{Cl}}^{-}$ form and 0.5 M CuCl_2_ in 5 M NaCl, the quantitative yield was registered after many hours (after 2 h of reaction somewhat above 90% of the functional groups were transformed into the ${\mathrm{CuCl}}_{4}^{2-}$ form).

The moist samples M/${\mathrm{CuCl}}_{4}^{2-}$ and G/${\mathrm{CuCl}}_{4}^{2-}$ coming from the transformation of the starting anion exchangers (M and G) [65,66] were intermediate products, which underwent various transformations. The peculiar affinity of the ${\mathrm{CuCl}}_{4}^{2-}$ ion towards the anion exchanger functional groups (strong bonding in a concentrated chloride solution and its absence in a dilute solution) was exploited to deposit various copper species in their matrix (17):



(17)

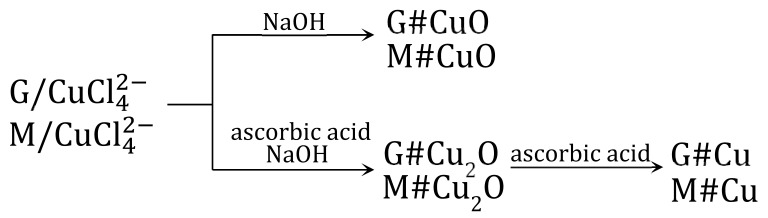




### 3.5. M#CuO and G#CuO Obtained via Anion Exchangers in the $CuCl_{4}^{2-}$ Form

To precipitate CuO within the macroporous resin phase [65], the (wet) intermediate product M/${\mathrm{CuCl}}_{4}^{2-}$ was introduced into the 1 M NaOH solution (18):

(18) $${\left(\left[P\right]-N^{+}{\left({\mathrm{CH}}_{3}\right)}_{3}\right)}_{2}{\mathrm{CuCl}}_{4}^{2-}+2{\mathrm{OH}}^{-}\to 2\left[P\right]-N^{+}{\left({\mathrm{CH}}_{3}\right)}_{3}{\mathrm{Cl}}^{-}\#\mathrm{CuO}+2{\mathrm{Cl}}^{-}+H_{2}O$$

In the course of 24 h of shaking, the resin sample changed its colour to blue (in a few minutes Cu(OH)_2_ was precipitated), then to black (in a few hours Cu(OH)_2_ was transformed to CuO). The product, M#CuO, contained 11.5 wt% of Cu, that is it contained two times more CuO than a similar product obtained by the indirect method (7). The distribution of the inorganic load within the porous matrix of the polymer beads was atypical. CuO was situated close to the surface of the beads, where it reached a Cu concentration of 55%, while the inner parts of the beads contained only an exceedingly small amount of evenly scattered CuO.

In the first study on the introduction of CuO into the matrix of a gel-like anion exchanger, probably via the tetrachlorocuprate ionic form (4.8 wt% of Cu), the means of synthesis and main product data were not reported in detail [67]. Based on our previously described results on M#CuO synthesis [65], we analogically introduced CuO into an anion exchanger gel-like structure by shaking for 24 h the (wet) intermediate product G/${\mathrm{CuCl}}_{4}^{2-}$ with a 1 M NaOH solution [66]. Comprehensive investigation using a gel-like anion exchanger in different ionic forms (${\mathrm{OH}}^{-}$ or ${\mathrm{Cl}}^{-}$) and different reaction media (5 M NaCl or 5 M HCl) showed that the G#CuO samples contained up to 8.8 wt% of Cu, but that the individual results did not fully reflect the degree of overreaction. Interestingly, the G#CuO samples from the HCl medium weighed about 6–7% less than the substrate (despite its high deposit content and the transition from the ${\mathrm{OH}}^{-}$ form into the ${\mathrm{Cl}}^{-}$ form), whereas the sample from the NaCl solution weighed 16–17% more than the host material. The cause of the reduction in the mass of G#CuO from the HCl medium was the decrease in the hydration of the functional groups when the ${\mathrm{OH}}^{-}$ form was replaced by the ${\mathrm{Cl}}^{-}$ form.

As the commercial anion exchanger gel-like structure occurred in the ${\mathrm{OH}}^{-}$ form, this made it possible to obtain an unusual product, i.e., G#${\mathrm{Cu}}_{2}{\left(\mathrm{OH}\right)}_{3}\mathrm{Cl}$, and G#CuO with a 25% higher CuO content (12.2 wt% of Cu) than previously [66]. At the beginning of the reaction G/${\mathrm{OH}}^{-}$ with 0.5 M CaCl_2_ in 5 M NaCl, a vivid green basic copper(II) chloride deposit appeared inside the beads:

(19) $$2\left[P\right]-N^{+}{\left({\mathrm{CH}}_{3}\right)}_{3}{\mathrm{OH}}^{-}+{\;\mathrm{CuCl}}_{4}^{2-}\to {\left(\left[P\right]-N^{+}{\left({\mathrm{CH}}_{3}\right)}_{3}\right)}_{2}{\mathrm{CuCl}}_{4}^{2-}+2{\mathrm{OH}}^{-}$$

(20) $$\left[P\right]-N^{+}{\left({\mathrm{CH}}_{3}\right)}_{3}{\mathrm{OH}}^{-}+{\;\mathrm{Cl}}^{-}\to \left[P\right]-N^{+}{\left({\mathrm{CH}}_{3}\right)}_{3}{\mathrm{Cl}}^{-}+{\;\mathrm{OH}}^{-}$$

(21) $$2{\mathrm{CuCl}}_{4}^{2-}+3{\mathrm{OH}}^{-}\to {\mathrm{Cu}}_{2}{\left(\mathrm{OH}\right)}_{3}\mathrm{Cl}\;+7{\mathrm{Cl}}^{-}$$

When the reaction medium was alkalized using a NaOH solution, the resin grains became black and the G#CuO obtained in this way contained an extra amount of CuO produced as a result of the decomposition of the ${\mathrm{Cu}}_{2}{\left(\mathrm{OH}\right)}_{3}\mathrm{Cl}$:

(22) $${\mathrm{Cu}}_{2}{\left(\mathrm{OH}\right)}_{3}\mathrm{Cl}\;+{\;\mathrm{OH}}^{-}\;\to \;2\mathrm{CuO}\;+2H_{2}O\;+{\mathrm{Cl}}^{-}$$

Detailed study of controlled and limited alkalization of the reaction medium resulted in copper rich G#${\mathrm{Cu}}_{2}{\left(\mathrm{OH}\right)}_{3}\mathrm{Cl}$ as a separate product (atacamite is insoluble in water and stable in neutral media).

It is worth emphasizing that G#CuO and G#${\mathrm{Cu}}_{2}{\left(\mathrm{OH}\right)}_{3}\mathrm{Cl}$ were atypical as regards the large amount of deposit they contain (up to 15.0 wt% CuO), as well as the peculiar distribution of the deposit into the grains. The accumulation of so much inorganic deposit, which in addition was non-uniformly distributed into the grains, resulted in very interesting phenomena on their surface.

### 3.6. M#CuO/FeO(OH) Obtained via Anion Exchangers in the $CuCl_{4}^{2-}$/$FeCl_{4}^{-}$ Form

Based on the previous procedure for introducing CuO into the anion exchange matrix [65] and a similar procedure using FeO(OH) [68], HIXs with mixed bimetal oxides were obtained [69]. Owing to the affinity of anion exchangers for the ${\mathrm{CuCl}}_{4}^{2-}$ ion, and also for the ${\mathrm{FeCl}}_{4}^{-}$ ion, and also the possibility of precipitating the respective oxides CuO and FeO(OH) after alkalization of the reaction medium, hybrid ion exchangers could be obtained containing Fe(III)/Cu(II) binary oxides. Given that the procedure for obtaining this type of material consists of several stages and quite complicated, and a gel-like anion exchanger has a compact polymer matrix that is difficult to access for large ions as ${\mathrm{CuCl}}_{4}^{2-}$ and ${\mathrm{FeCl}}_{4}^{-}$, in our research on the synthesis of this group of HIXs we focused on the use of an anion macroreticular structure as a host material. Thanks to this structure, the large-sized ions used as the substrates could move freely in the resin into the centre of the beads.

The FeO(OH) deposit was Introduced Into the matrix of the anion exchanger as a result of the following transformations:

(23) $${\mathrm{FeCl}}_{3}+{\mathrm{Cl}}^{-}\;\to {\mathrm{FeCl}}_{4}^{-}$$

(24) $$\left[P\right]-N^{+}{\left({\mathrm{CH}}_{3}\right)}_{3}{\mathrm{Cl}}^{-}+{\;\mathrm{FeCl}}_{4}^{-}\to \left[P\right]-N^{+}{\left({\mathrm{CH}}_{3}\right)}_{3}{\mathrm{FeCl}}_{4}^{-}+{\;\mathrm{Cl}}^{-}$$

(25) $$\left[P\right]-N^{+}{\left({\mathrm{CH}}_{3}\right)}_{3}{\mathrm{FeCl}}_{4}^{-}+3{\mathrm{OH}}^{-}\to \left[P\right]-N^{+}{\left({\mathrm{CH}}_{3}\right)}_{3}{\mathrm{Cl}}^{-}\#\mathrm{FeO}\left(\mathrm{OH}\right)+3{\mathrm{Cl}}^{-}+H_{2}O$$

In previous studies, we found that in 0.5 M FeCl_3_ in 5 M HCl solution, the ${\mathrm{FeCl}}_{4}^{-}$ ions can be quantitatively and over a short time bound by the functional groups of the anion exchanger [68], similarly to the ${\mathrm{CuCl}}_{4}^{2-}$ ions in 0.5 M CuCl_2_ in 5 M HCl solution, or in 0.5 M CuCl_2_ in 5 M NaCl solution [65]. The aim of subsequent studies was to introduce into the anion exchanger the composition of iron(III)/copper(II) oxides with a Fe/Cu molar ratio of 2:1, characteristic for copper ferrite. This was not an easy task since the ions had different valences and differed in their affinity to the anion exchanger functional groups. It turned out that the ${\mathrm{FeCl}}_{4}^{-}$ ions, even though monovalent, exhibited greater affinity for the anion exchanger functional groups than the divalent ${\mathrm{CuCl}}_{4}^{2-}$ ions.

The oxide deposit was introduced into M matrix in three ways, whereby HIXs were obtained that differed in oxide content, the mole ratio of the oxides, and their distribution in the matrix of the anion exchanger [69]. The following procedures were used: (1) both ${\mathrm{FeCl}}_{4}^{-}$ and ${\mathrm{CuCl}}_{4}^{2-}$ ions were introduced into the ion exchanger and then through alkalization the two oxides (FeO(OH) and CuO) were simultaneously precipitated, (2) first ${\mathrm{CuCl}}_{4}^{2-}$ ions were introduced into the ion exchanger, subsequently some of them were replaced with ${\mathrm{FeCl}}_{4}^{-}$ ions and then through alkalization the two oxides (FeO(OH) and CuO) were simultaneously precipitated, (3) first ${\mathrm{FeCl}}_{4}^{-}$ ions were introduced into the ion exchanger and through alkalization FeO(OH) was precipitated, then ${\mathrm{CuCl}}_{4}^{2-}$ ions were introduced into the ion exchanger and through alkalization CuO was precipitated. Regardless of the method of conducting the reaction, HIXs rich in oxides were obtained. The product with the highest content of both oxides, e.g., 13.2 wt% of Fe and 8.8 wt% of Cu, at Fe/Cu = 1.7 was obtained using procedure (3). The obtained HIXs showed magnetic properties: ferromagnetic (procedure 1 and 2) or paramagnetic (procedure 3). The samples sintered at 900 °C and 1300 °C contained CuFe_2_O_4,_ which proved that a ferrite could form from the deposit after a solid-state reaction at high temperatures.

### 3.7. M#Cu_2_O and G#Cu_2_O Obtained via Anion Exchangers in the $CuCl_{4}^{2-}$ Form

Our previous work on the introduction of Cu_2_O into the anion exchanger by the indirect method [61,64] showed that retaining the forming oxide in the resin phase posed a major challenge. This was only partially achieved and only in specific conditions. In order to obtain M#Cu_2_O and G#Cu_2_O with an elevated Cu_2_O content, we tested the method based on quantitative transformation of the functional groups of the anion exchanger in the tetrachlorocuprate ionic form, followed by the reduction of this ion to Cu_2_O [70]. Due to the anionic form of the cuprous oxide precursor, the proposed method might lead to obtaining HIXs with larger Cu_2_O content than by reduction of the Cu(OH)_2_ (9) [61] or Cu_4_(OH)_6_SO_4_ (14) [64] deposit. The selection of the reducer and reaction conditions was crucial so that the Cu_2_O formed remained in the resin phase to as great a degree as possible. Previous studies have shown that redox reactions pose a challenge in the synthesis of materials such as HIXs, especially when the reducer acts slowly.

To precipitate Cu_2_O within the macroporous anion exchanger, the (wet) intermediate product M/${\mathrm{CuCl}}_{4}^{2-}$ (from 0.5 M CuCl_2_ in 5 mol NaCl, or 0.5 M CuCl_2_ in 5 M HCl) was put into contact with a reducer solution, which was ascorbic acid (AA) or glucose (GL) [70]. To ensure favorable conditions for the reduction and to prevent copper passing into the aqueous phase, besides the reducer, the reaction medium contained NaOH in an amount ensuring a high pH at each of the reaction steps (including after the neutralization of the concentrated HCl solution remaining in the porous structure of the resin after filtration). Studying the influence of numerous factors on the course of the reaction, it was found that much more Cu_2_O was deposited in the resin when an ascorbic acid solution was used:



(26)

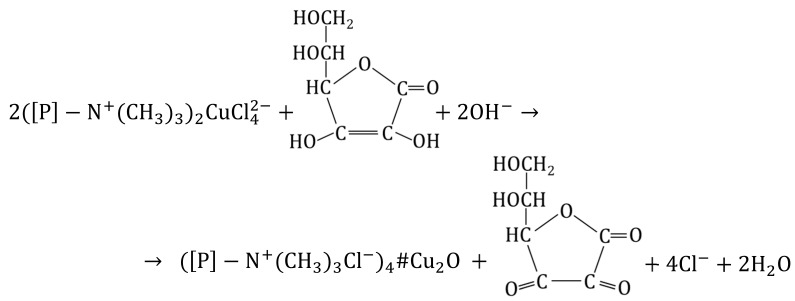




Unexpectedly, it turned out that the result was influenced by the origin of the M/${\mathrm{CuCl}}_{4}^{2-}\;$ sample from a 5 M HCl solution or a 5 M NaCl solution. The copper content in the M#Cu_2_O varied depending on the reaction medium in the first stage of synthesis (HCl vs. NaCl), as well as on the type of reducer in the second stage of synthesis (AA vs. GL) in the following order: HCl/AA > NaCl/AA > > > NaCl/GL > HCl/GL. The highest degree of Cu_2_O introduction into M (almost 90% conversion, 8.65 wt% of Cu) was achieved in the case of the sample derived from the HCl medium and reduced with AA (only 4.0 wt% of Cu was introduced into M using GL). It was interesting that the NaCl/AA sample vs. the HCl/AA sample (reduced with the same reducer in the same conditions, 0.25 M AA in 1 M NaOH), contained only 6.3 wt% of Cu. It was found that ascorbic acid immediately reduced the ${\mathrm{CuCl}}_{4}^{2-}$ ion to Cu_2_O, whereby the reduction product remained in the resin phase. In the case of glucose, the reduction proceeded slowly, whereby a substantial amount of Cu_2_O passed outside the resin (into the water phase).

When the G/${\mathrm{CuCl}}_{4}^{2-}$ sample from the HCl medium was reduced with an ascorbic acid solution, the G#Cu_2_O contained about 6.6 wt% of Cu, whereas the sample reduced with glucose contained only 2.5 wt% of Cu. It is interesting that when the sample came from the NaCl medium, and was reduced with ascorbic acid solution, it contained over 7.0 wt% of Cu. Thus, for G/Cu_2_O, the origin of the sample (formation of G/${\mathrm{CuCl}}_{4}^{2-}$ in 5 M NaCl or 5 M HCl) had an insignificant influence on the reaction yield. About 25% less Cu_2_O was deposited in the G matrix vs. the M matrix, which was due to both the incomplete conversion of the functional groups into the ${\mathrm{CuCl}}_{4}^{2-}$ form and the gel-like structure slowing down the reduction.

### 3.8. M#Cu and G#Cu Obtained by Reduction of M#Cu_2_O and G#Cu_2_O

Considering that ascorbic acid easily reduces Cu_2_O to metallic copper in (bulk) aqueous media at pH < 7:



(27)

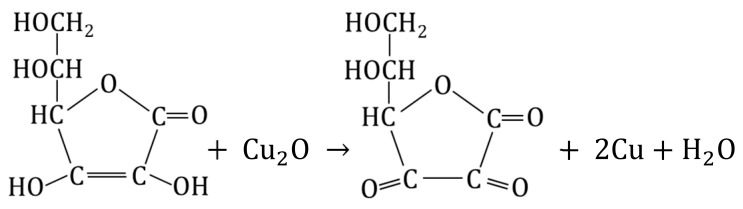




We carried out a similar transformation inside anion exchangers to convert M#Cu_2_O to M#Cu and G#Cu_2_O to G#Cu [71]. The Cu_2_O in the polymeric matrices was in the form of spherical particles occurring singly (G/Cu_2_O) or in the form of bracelet-like clusters (M/Cu_2_O). The particles were almost ideally distributed through the volume of the beads. The remarkably high Cu_2_O content and the advantageous distribution of the deposit throughout the volume of the resin beads make M#Cu_2_O and G#Cu_2_O ideal substrates for synthesizing even more unique materials, such as metallic copper-doped anion exchangers [70]. Two reaction media with pH < 7 but differing in acidity were evaluated. These were a solution of ascorbic acid alone (1 M ascorbic acid, pH~2), and a solution of ascorbic acid neutralized with an equimolar amount of NaOH (1 M sodium ascorbate, pH~6). As the result of a single-step transformation of preformed Cu_2_O particles conducted using an eco-friendly reducer and under ambient conditions, macroporous and gel-type HIXs were obtained with a copper content of above 7.0 and 5.0 wt% respectively. It turned out that regardless of the reaction medium and reduction conditions (3 h at 50 °C or 24 h at 20 °C) all products after reaction contained a large amount of copper, with the degree of conversion amounting to 85–95%. The actual Cu content in the sample did not fully reflect the degree of transformation of Cu_2_O into Cu^0^ because the mass of the products was up to 25% greater than that of the initial samples owing to the transformation of the functional groups from the hydroxyl to ascorbate form. The ascorbate form of the functional groups was advantageous due to its antioxidant properties, which protected the newly formed Cu^0^ from undesirable oxidation.

As the transformation of the Cu_2_O particles into the Cu^0^ particles took place in atypical conditions, that is in the solid phase of the anion exchangers, it had to be investigated how, as a result of the chemical reaction, the introduced deposit changed into the deposit characterized by a different chemical structure and different physicochemical properties, and which after the reaction ought to remain in the resin phase. We worked out this issue in detail [71], taking into account the type of porosity of the supporting polymer and its integrity after drying, the size, shape and distribution of the preformed Cu_2_O particles in the beads of the starting material, the drying operations before the reduction reaction (thermally dried substrate vs. wet substrate) and the post-reduction reaction (thermally dried final product vs. freeze dried final product).

### 3.9. Comments on Direct Synthesis

Summing up this part of the synthesis, it can be stated that with the use of both anion exchangers (M and G) with the functional groups in the ${\mathrm{CuCl}}_{4}^{2-}$ form, only some HIXs (containing CuO, Cu_2_O, Cu) can be produced, but they contain much more deposit than analogous composites obtained by the indirect method. Unfortunately, HIXs containing Cu(OH)_2_, Cu_4_(OH)_6_SO_4_, Cu_4_O_3_ cannot be obtained by the direct method, however, HIXs containing mixed bimetal oxides, for example Fe(III)/Cu(II) binary oxides, can be obtained. A fundamental issue in direct synthesis of HIX is to ensure such reaction conditions that the expected decomposition products of ${\mathrm{CuCl}}_{4}^{2-}$ ions remain in the resin phase, which means that the reaction that leads to the precipitation of the deposit must occur quickly. In an NaOH solution, the ${\mathrm{CuCl}}_{4}^{2-}$ ions immediately decomposed, and the forming Cu^2+^ ions diffused from the inner to the outer part of the beads due to electrostatic repulsion by positively charged functional groups, with the macroporous structure of the resin favoring this displacement. However, in an NaOH/reducer solution, decomposition of the ${\mathrm{CuCl}}_{4}^{2-}$ ions causes the formation of unstable Cu^+^ ions, which before being pushed outward by the functional groups, precipitate rapidly as Cu_2_O. So, M#CuO and G#CuO obtained by the direct method contain a deposit close to the surface of the beads, M#Cu_2_O and G#Cu_2_O contain a deposit dispersed throughout the volume of the beads, whereas M/Cu and G/Cu contain a deposit whose location results from the substrate.

## 4. Properties and Applications of Copper-Containing HIXs

### 4.1. Influence of Copper Deposit on the Resin Phase

**Figure 3:**
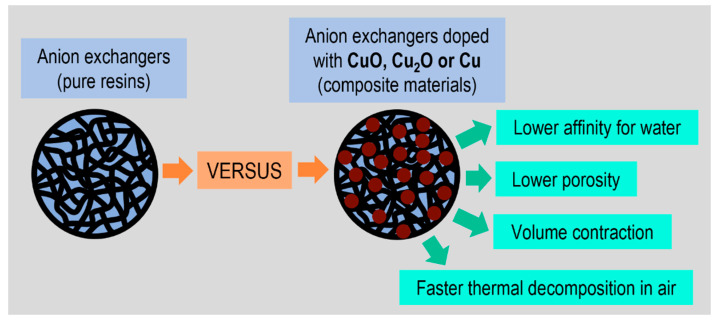
Influence of the copper compounds deposit on the physicochemical properties of polymeric support.

When copper-containing fine particles are immobilized in the resin phase, such composites exhibit a lower affinity for water compared to pure resin (Figure 3). This is mentioned so as to explain the results of the thermogravimetric analysis, as the first step in the thermal decomposition of such materials as ion exchangers and hybrid ion exchangers is dehydration [72]. This change in the TG/DTG curves corresponds to the sample mass decrement due to water evaporation. Typically, classic ion exchangers, cation exchangers and anion exchangers have ionogenic, polar functional groups surrounded by water molecules, which is referred to as solvation. They contain about 15.0% hygroscopic water. Thermogravimetric analyses of hybrid polymers with different copper deposits, that is CuO, Cu_2_O and Cu^0^, have shown a strong relationship between the copper oxidation state and the content of surface-bound water. This value decreases along with the decrease in Cu oxidation as follows: M (15.4%) > M#CuO (13.0%) > M#Cu_2_O (10.7%) > M#Cu (7.2%) and G (15.2%) > G#CuO (13.3%) > G#Cu_2_O (9.4%) > G#Cu (4.3%). This was particularly evident in the case of G#Cu, which anomalously contains little hygroscopic water, with rare amounts as low as around 4%. So, Cu^0^ in the polymer matrix is a much stronger water repellent compared to Cu_2_O and CuO. There is no doubt than the decrease of the hygroscopic water content in the studied composites is caused by the presence of the metallic copper/copper compound in them. The same host materials (both structure anion exchangers), into which matrix we introduced ferric oxide particles, contained about 14.5% of bound water (ferric oxides occur in hydrated form, while metallic copper and copper compounds exhibit no affinity or low affinity for water) [73].

The limited content of hygroscopic water in the studied materials is related to another interesting property, namely the unprecedented low porosity of the composites’ macroporous structure (the porosity applies only to the resins’ macroporous structure, which contains artificial open pores in the form of channels created during polymerization). Through comprehensive experimentation using the N_2_ adsorption-desorption method and mercury intrusion porosimetry (determination of surface area, pore volume, average pore diameter, pore surface area and total porosity), we demonstrated that the dispersion of Cu^0^, Cu_2_O and CuO particles in the matrix of the macroporous anion exchanger results in an increase in bulk density and practically a loss of porosity in the thermally dried composite materials in relation to the pure resin (M) [74]. The thermally dried composite material (above all M#Cu, but also M#Cu_2_O) not only shrank because of water loss, but its pores also narrowed/collapsed, which resulted in volume contraction. The decay of the porous structure resulted from the surface morphology of Cu^0^, Cu_2_O and CuO particles, their hydrophobicity and propensity to agglomeration. In contrast to thermal drying, composites that were freeze dried retained the porous characteristics typical for a macroporous anion exchanger.

### 4.2. Thermal Properties

Thermal analysis, including thermogravimetry (TG) and differential thermogravimetry (DTG) is a basic method that provides information on the thermal behavior/thermal resistance of ion exchangers. We have extended the scope of this method by subjecting samples of HIXs to thermal analysis so as to compare the results with those of the starting materials (the anion exchangers with no copper deposit). The aim of our investigations was to clarify the role of the copper deposit (copper compounds show catalytic properties), and more precisely to determine the effect of the oxidation state of the copper atom in the deposit on the thermal properties of the composite materials.

The investigations involved 10 composite samples and were performed in air and in nitrogen, as combustion and pyrolysis are the two main decomposition techniques used for the examination of ion exchangers. The results, i.e., TG/DTG curves with the complete set of numerical data (characterizing the transformation, their temperature range, the temperature at which there is the maximum decomposition rate, mass change, end temperature and residual mass) provided a basis for comprehensive analysis of the thermal decomposition of HIXs [73,75]. It has been shown that some cupric compounds, cuprous oxide and metallic copper deposited in the skeleton of both types of anion exchanger significantly alter the results of the thermal analysis in comparison with those for pure resin. The differences resulted from the process end temperature, the acceleration of the decomposition steps, the amount of carbonizate in the post-pyrolysis residue, and the composition of the inorganic phase. After transformation in air, the residues contained CuO, whereas those after transformation in N_2_ contained metallic copper and char from the resin phase. One of the more notable achievements of our research consists of demonstrating that in N_2_, the transforming polymeric phase can reduce the inorganic deposit, whereby a larger part of it than in the case of pure resin condenses into products with large non-volatile particles. The oxidation state of the copper atom in the deposit has an increasing effect on the amount of char that forms. The decomposition processes allowed for a sequential decrease (in stages) in the mass/volume of HIX, and its transformation into an innovative copper/carbon char composite. The solid residues after pyrolysis for the M#CuO, M#Cu_2_O, M#Cu and M form the series 34.1, 31.2, 28.5 and 11.2% respectively, while the series for G#CuO, G#Cu_2_O, G#Cu and G is 39.5, 26.8, 24.3 and 14.7%. Instead of synthetic carbon char prepared on the basis of an anion exchanger alone, by pyrolysis of HIX one can obtain metal-doped carbon char possessing a far more diverse composition and properties.

### 4.3. Ability to Remove As(III) from Water

**Figure 4:**
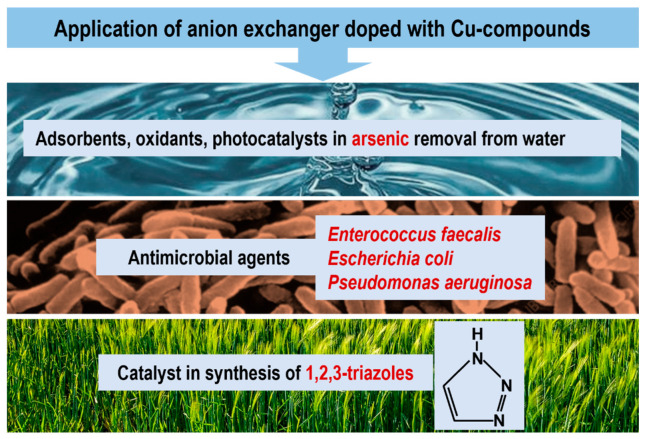
Application of anion exchangers doped with copper compounds.

Removal of arsenic from water to below 10.0 µg As/dm^3^ is one of the major global environmental problems. Arsenic species are extremely harmful to the human body and occur in elevated concentrations in natural water used as drinking water by the inhabitants of many regions of the world. In order to reduce the elevated levels of arsenic in water, various sorbents, including parent metal oxides and also hybrid ion exchangers containing dispersed metal oxides (FeO(OH), MnO_2_, ZrO_4_), have been extensively investigated due to their high affinity and selectivity toward metalloids such as As(III) and As(V). Considering that parent CuO and CuO/FeO(OH) are promising materials as arsenic sorbents, and considering the photocatalytic properties of parent Cu_2_O, we checked the suitability of our materials in this area (Figure 4). Their beneficial physical form (fine particles immobilized within a mechanically and chemically stable, porous and permeable support) allows many technical problems resulting from the use of NPs alone (without polymeric carrier) to be overcome. For the study, we selected M#CuO, M#CuO/FeO(OH), and also M#Cu_2_O.

First, M#CuO was used for As(III) removal from water [76]. In the process of removing arsenic from water, the basic issue is the oxidation of As(III) to As(V). Our studies included batch kinetic and equilibrium experiments, the influence of the pH, the regeneration of spent adsorbent and the column process on As(III) adsorption. Due to the combination of both components, CuO and the anion exchanger, arsenite removal proceeded in two steps: oxidation to arsenate on the CuO surface, followed by an ion exchange reaction involving electropositive functional groups of M. In comparison to HIXs containing other metal oxides, M/CuO exhibited an average adsorption capacity of 6.6 mg As/g, but very good kinetic parameters as a result of the beneficial location of CuO in the outer parts of the beads, which diminished the influence of intraparticle diffusion on the adsorption rate. The very good kinetic characteristics enabled the process to be performed effectively under a dynamic regime.

Extending the previous research, in the next study into As(III) removal from water, three types of M#CuO/FeO(OH) were used with different amounts and distribution of Cu(II)-Fe(III) binary oxides within the polymer beads [77]. An adsorption capacity as high as 94.4 mg As/g was demonstrated in the material containing the inorganic deposit in the outermost parts of the polymer beads through significant improvement in the kinetic parameters of the process. The adsorption was effective over a wide pH range, and was selective in the presence of interfering ions. The performance of the hybrid polymer was confirmed in the column process, which enabled As(III) and As(V) concentrations to be lowered from 500 µg/dm^3^ to below 10 µg/dm^3^ in a solution with a composition similar to ground water. The breakthrough point of the bed was reached after almost 1850 bed volumes of the solution had passed through the column. After use, the adsorbent was easily regenerated, which allowed multiple cycles of arsenic adsorption-desorption to be conducted with high efficiency. The research showed that As(III) was mainly adsorbed on the surface of Cu-Fe oxides, followed by its oxidation to As(V) and the formation of stable complexes on their surface. In the oxidation reaction, the metal oxides acted as catalysts and adsorbents, while the oxidant was probably the oxygen dissolved in the solution.

In the next research into As(III) removal from water, we explored the photocatalytic activity of M#Cu_2_O, and explained the mechanism of its action in water purification processes [78]. The adsorption studies were conducted in the dark, UV-Vis irradiation, and Vis irradiation, and in aerobic and anoxic conditions. The process conditions—light irradiation, presence of oxygen and accompanying processes such as photocorrosion and ageing during storage and during the adsorption process—influenced the mechanism and the efficiency of arsenic removal. The study showed that the mechanism of As(III) oxidative adsorption is based on the coupling of several pathways: photocatalytic oxidation involving Cu_2_O as a photocatalyst, chemical oxidation on the surface of CuO as a result of the ageing process, and finely photochemical oxidation of As(III) in the solution under UV light irradiation, followed by subsequent adsorption of arsenates in the functional groups of the anion exchanger.

### 4.4. Ability to Remove Phosphate from Water

Wastewater that contains phosphate contaminate natural aquatic environments and are the main cause of eutrophication in rivers and lakes. To limit the release of phosphate into natural systems, new ways of its selective removal during wastewater treatment are required. The method that used the oxides of polyvalent metals dispersed within the anion exchanger turned out to be noteworthy because these oxides in their native form exhibit adsorptive selectivity towards ${\mathrm{HPO}}_{4}^{2-}$ and $H_{2}{\mathrm{PO}}_{4}^{-}$ as a result of ligand sorption through the formation of inner-sphere complexes via Lewis acid-base interactions. To remove phosphate from aqueous media, three HIXs containing hydrated ferric oxide (7.62 wt% of Fe), hydrated zirconium oxide (6.74 wt% of Zr) and hydrated copper oxide (4.82 wt% of Cu) were used, reaching a maximum adsorption capacity amounting respectively to 111.1, 91.74 and 74.07 mg P/g [67]. When the hybrid materials were tested in a solution also containing sulphate (as a competing ion) the maximum capacity strongly decreased and was respectively 18.5, 19.0, and 6.0 mg P/g. Comprehensive studies using a synthetic mono-ionic solution of phosphate, a binary solution of phosphate with sulphate, and two real wastewaters showed a decreased usefulness of G#CuO compared to HIXs with FeO(OH) and ZrO_2_ particles. According to the authors, the low capacity of G#CuO for phosphate adsorption could result from the too short metal-oxygen bond length, since 1.91 Å could fall below a critical bond length needed to effectively adsorb phosphate from a highly mixed and complicated matrix such as wastewater (the adsorption process depends on the feasibility of releasing the surface functional groups -H_2_O and -OH, and this is directly related to the metal-oxygen bond length). It can be assumed that the low capacity of G#CuO for phosphate adsorption could also result from the lower hydration of CuO (compared to FeO(OH) and ZrO_2_) as its surface has a narrower positively charged pH range, which worsens the electrostatic attraction of anionic adsorbates.

### 4.5. Antimicrobial Properties

Metallic copper (which has been used to sanitize water for hundreds of years) and its compounds such as copper oxides, copper hydroxide and basic copper salts, exhibit high biocidal activity against a wide range of microorganisms, both obligatory and opportunistic pathogens. As the search for new antimicrobial agents is a high priority in today’s world due to the spread of pathogens and the necessity to minimize the risk of their occurrence, it was justified to investigate the antimicrobial activity of our composites considering their favorable chemical composition. It should be emphasized that they differed significantly from low-molecular-weight and nano-sized antimicrobial agents, and that no HIXs have been subjected to this type of research so far.

In the preliminary study, four selected composite materials containing Cu(II) compounds and both supporting materials were studied for their potential uses against the opportunistic, Gram-positive *Enterococcus faecalis*, an example of bacteria from among significant human pathogens [79]. The study examined the influence on biological activity of all components of the hybrid polymer, such as type and morphology of Cu deposit, its distribution within the beads, Cu^2+^ release to the aqueous phase, as well as the different structure of the supporting material (M or G) containing trimethyl ammonium functional groups. It has been shown that all the studied materials exhibited antibacterial activity in the following order M#Cu(OH)_2_ > G#Cu_4_(OH)_6_SO_4_ > M#CuO = G#CuO > M > G, and that the morphology and distribution of cupric deposit within the resin skeleton was crucial for biocidal properties, while the chemical composition of cupric compounds and the amount of copper deposit had a negligible effect. M#Cu(OH)_2_ showed the best biological efficiency with complete killing (7log) of *E. faecalis* at an 8-fold lower dose in comparison with the other materials. Meanwhile, M#Cu(OH)_2_ and G#Cu_4_(OH)_6_SO_4_ contained cupric deposit in the form of agglomerated particles with an irregular and roughened surface, and the thick, compact and homogeneous CuO layer of both M#CuO and G#CuO was less accessible to bacteria, resulting in worse biocidal properties. The decrease of survival after exposure to increasing doses of M and G indicated that both supporting materials also contribute to the antibacterial activity of the examined composites.

Gram-positive *Enterococcus faecalis* and Gram-negative *Escherichia coli* were then chosen as common opportunistic pathogens to be inactivated by G#Cu_2_O [64]. The synergistic effect of both components against Gram-positive *enterococci* was again demonstrated, as the anion exchanger not only provided a supporting matrix for Cu_2_O, but also enhanced the antibacterial performance of this composite. A 100% reduction of viable cells was observed from 5 log_10_ CFU/mL to 0 after 24 h of incubation with 128 of An#Cu_2_O. In similar studies, an increase in the mass of the anion exchanger alone (G) also led to a successive reduction in the number of viable cells of *enterococci*, although to a lesser extent. In contrast, in the case of *E. coli* there was no difference in the bacterial effect of the anion exchanger with and without Cu_2_O deposit. This clearly indicated the bactericidal effect of the host material itself (due to the presence of quaternary ammonium groups), while the inorganic deposit only insignificantly strengthened the antibacterial activity of the composite material.

To extend the research and gain a better insight into the mechanism of the bacterial activity of the HIXs containing Cu_2_O, we used M#Cu_2_O and G#Cu_2_O obtained via ${\mathrm{CuCl}}_{4}^{2-}$ ions (composites richer in Cu_2_O) against *Enterococcus faecalis* (Gram-positive) and *Pseudomonas aeruginosa* (Gram-negative), which represented two groups of opportunistic bacteria [80]. In contrast to previous studies based on determining MBC values for planktonic cells of bacteria, in this work another stage of research is presented on the factors controlling biocidal activity, based on a combination of chemical and biological tests. The diverse biocidal activity of the two HIXs against both bacteria was confirmed. The differences in this activity were more due to the differences between the bacteria (Gram-positive vs. Gram-negative) and less to the differences between the materials (macroporous vs. gel-like matrix structure). The variances resulting from various strategies of bacterial cell resistance to heavy metals present in the environment became apparent during kinetic studies carried out in different conditions—static vs. dynamic culture, and culture medium—natural water vs. Muller-Hinton broth (the composition of which is crucial to the release of copper ions to the liquid phase and copper complexation in the solution). Confocal microscopic studies confirmed that the positively charged surface of the polymeric beads impeded biofilm formation because of contact-killing, while the released Cu^2+^ showed high biocidal activity against planktonic bacteria present in the bulk solution. Due to the application of non-standard procedures, this study revealed more precisely the mechanism of the antibacterial activity of the multifunctional heterogeneous composites—cuprous oxide-containing anion exchangers.

### 4.6. Catalytic Properties

As parent copper nanoparticles are a new class of heterogeneous catalysts in various chemical transformations, a polymer-supported copper catalyst was obtained using a macroporous anion exchanger as the host material. Cu_2_O NPs with a diameter of 5–7 nm were immobilized onto the M surface (1.08% wt% of Cu) using the indirect method of synthesis with the use of an anion exchanger in the formate (${\mathrm{HCOO}}^{-}$) form [62]. The composite with agglomeration-free uniformly dispersed Cu_2_O exhibited excellent catalytic performance in the synthesis of 1,2,3-triazole, and in the regioselective synthesis of diversely functionalized 1,2,3-triazoles via a three-component reaction of alkyl halide, terminal alkyne, and sodium azide (triazole-based dithiocarbamate belongs to antitumor drug molecules). Notable features were the aqueous reaction media, ambient reaction conditions, high yields, wide substrate scope, as well as easy recovery of the catalyst by filtration and its reusability. The catalytic system exhibited nearly double TOF (219 h^−1^, turnover frequency) compared to existing systems, which according to the authors was attributed to the availability of active Cu_2_O on the surface of the beads.

## 5. Conclusions

Composite materials that are anion exchangers with both a macroporous and gel-like structure, to which an inspiring and exciting element such as copper has been introduced, constitute a new and large group of hybrid ion exchangers exhibiting peculiar properties. Using simple synthesis pathways (aqueous media, ambient conditions, cheap and common reagents) enables the preparation of composites containing metallic copper, copper oxides, cupric hydroxide and cupric hydroxysalts. Taking into account the fact that the copper content in the composites (3.0–12.5 wt% of Cu) is connected with the ion exchange capacity of the supporting materials (the content of functional groups in both anion exchangers was around 3.3. meq/g), it can be said that the research succeeded in obtaining composite materials with a high degree of overreaction. The successful synthesis results stemmed mainly from the favorable properties of expected copper derivatives, which in established conditions easily, quickly, and in some cases quantitatively precipitated in the polymeric phase using as the copper precursor Cu^2+^ ions in the solution or ${\mathrm{CuCl}}_{4}^{2-}$ ions bonded by functional groups of the resin.

Comparison of the products by copper content (per mass unit) does not fully reflect the degree of transformation. There are transformations in which the mass of the resulting HIX is much less than the mass of the parent resin; as a result, the copper content in such a product seems to be too high. In general, the “Cu content in sample” parameter is affected by both the polymeric support and the deposit. This value depends primarily on the course of the reaction (reaction rate and deposit location in the resin phase). Secondly, it depends on the ionic form of the anion exchanger functional groups (the mass of ascorbate ions is much larger than the mass of hydroxyl ions). Thirdly, it depends on the hydration of the functional groups (depending on the ionic form, the functional groups are hydrated to varying degrees). Fourthly, it depends on the type of copper deposit; such deposit as metallic copper acts as water repellent (and other deposits do not exhibit such a property).

Copper-containing particles dispersed within the polymeric support modify its properties, leading to a tighter composite structure, decreasing the affinity toward water, resulting in a lower moisture content, and increasing the hydrophobic properties, thus enhancing antimicrobial activity. Various characteristic features of the materials described, as well as their specific properties, mean that they can be used in water purification processes for particular tasks, in which their multifunctionality is essential, i.e., the ability to act in many ways (as an adsorbent, oxidant, biocide or catalyst). This is due to the presence of trimethylammonium functional groups, the chemical composition of the inorganic phase and its arrangement in the resin beads.

In addition to the transformation presented in this work, there are numerous interesting preparative possibilities, for example, the sulfidation of copper oxides to obtain HIXs with Cu_x_S, obtaining HIXs with various compositions of binary oxides, as well as the discovery of new composites containing other transition metals that exhibit reactivity and physicochemical properties similarly to copper.

Materials already described, materials planned by us to obtain, and many more of this kind in future can be widely used due to the presence of copper atoms in the deposit; at the same time, each type of copper deposit brings to the products characteristic, specific and often separate properties (metallic copper is a reducer, CuO is a oxidant). The application of these materials may be associated not only with water purification but with many other areas where the presence of copper is crucial (antibacterial action, catalysis, photocatalysis, and others). It is hoped that these results will be an inspiration to solve important environmental research problems. First of all, it is about removing from water heavy metals, troublesome pollutants such as synthetic dyes and different non-biodegradable and bioaccumulative organic contaminants.

## Data Availability

Not applicable.
